# Posterior Ligamentous Complex Injuries of the Thoracolumbar Spine: Importance and Surgical Implications

**DOI:** 10.7759/cureus.18774

**Published:** 2021-10-14

**Authors:** Aren Joe Bizdikian, Rami El Rachkidi

**Affiliations:** 1 Department of Orthopaedics and Traumatology, Hotel-Dieu de France Hospital, Beirut, LBN

**Keywords:** thoracolumbar spine, minimally invasive spine surgery, spinal fixation without fusion, spinal fusion, spinal instability, spine trauma, posterior ligamentous complex injuries

## Abstract

The soft tissues surrounding the spine play a primordial role in its stability, the most important of which are located posteriorly and are deemed the posterior ligamentous complex (PLC). Injuries to the PLC in the setting of thoracolumbar trauma are often dreaded and little attention has been given to them in the management protocols of thoracolumbar trauma. This review aims to summarize and contextualize current concepts in PLC injuries of the thoracolumbar spine with the aim to provide a clear guide for clinical management.

Injuries to the PLC may be suspected on the clinical exam but are often missed, leading to serious complications, including instability and neurological compromise. The diagnosis is often made indirectly by spinal radiographs and CT-scanning or by direct visualization of soft tissues via magnetic resonance imaging. The latter remains the standard imaging modality and is mandatory for patients with a high suspicion of PLC injury. PLC injuries are associated with vertebral fractures and follow a progressive pattern of severity, depending on the mechanism of injury and extent of trauma. Surgical management is warranted, as PLC damage renders the spine unstable. Although fusion was once the standard of care and remains applicable for certain patients, recent endeavors of temporary spinal fixation without fusion are increasingly gaining traction in patients with PLC injuries.

In conclusion, PLC injuries are challenging as they are often missed, poorly understood, and are not easily managed. Proper diagnosis and management are crucial to avoid long-standing complications such as spinal instability. Considering the paucity of available data on such an important topic in thoracolumbar trauma, this review article aims to contextualize current concepts in PLC injuries in order to demystify this sparsely covered subject.

## Introduction and background

The spine serves many roles, including locomotion and the protection of the spinal cord, functions that would not be possible without stability, a feature enabled by the vertebrae themselves, surrounding ligaments, intervertebral discs, and facet joints [1]. Without stability, uneven forces would be exerted on the different spinal segments resulting in increased energy expenditure, general muscle fatigue, deterioration in the quality of life, potential spinal cord compromise, and secondary neurological deficits [2,3].

While much importance has been attributed to thoracolumbar fractures, data found on posterior ligamentous complex (PLC) injuries pales in comparison. Although many classification systems have been proposed for thoracolumbar trauma, PLC injuries have been superficially and sporadically addressed, and most studies lack granularity on the severity of this injury [4-8]. Similarly, spinal fracture treatment strategies have been thoroughly studied, but PLC injuries seem to lack clear-cut guidelines [9-12].

This review article aims to contextualize current concepts in PLC injuries in order to demystify this sparsely covered subject.

## Review

Descriptive and functional anatomy

**Figure 1:**
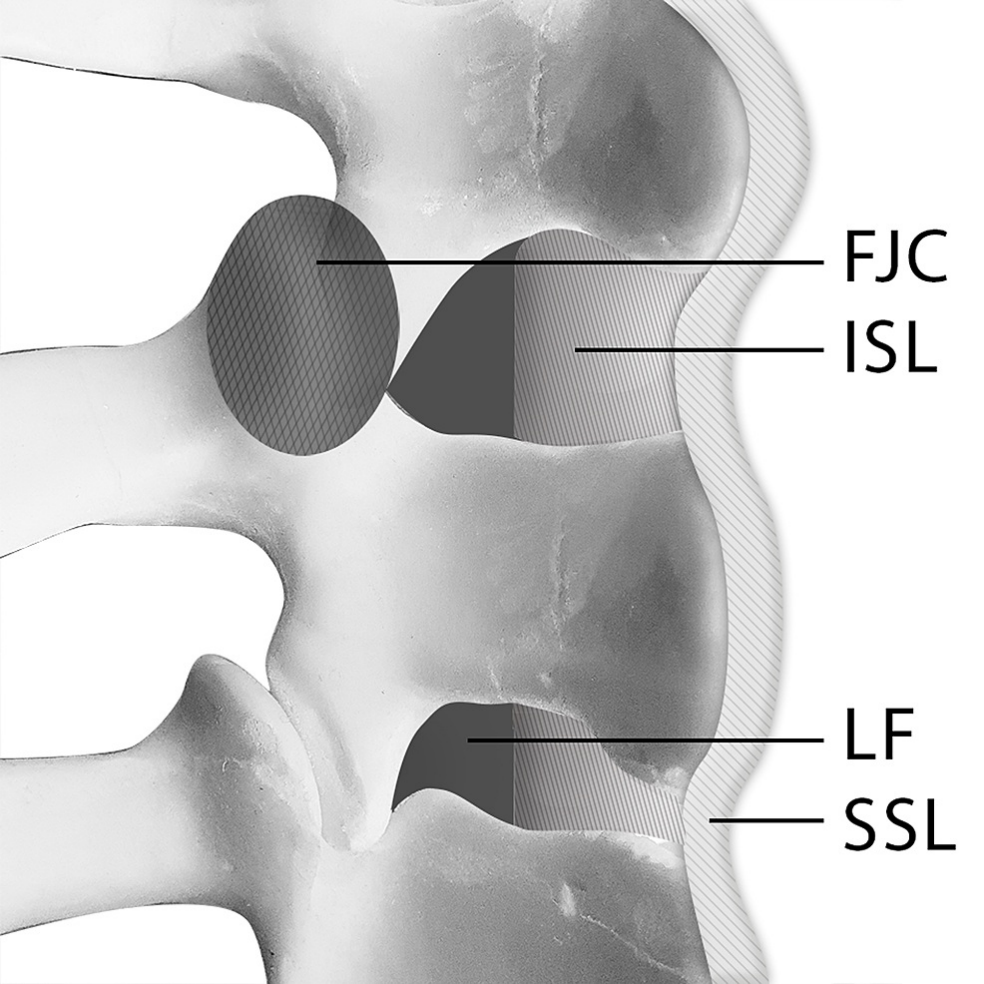
Posterior ligamentous complex (PLC): facet joint capsule (FJC), the supraspinous ligament (SSL), interspinous ligament (ISL), and ligamentum flavum (LF).

The PLC consists of multiple ligaments that span the posterior aspect of the spine. These ligaments, together with the posterior vertebral arches, make up the posterior osseoligamentous complex (Figure 1). The PLC is composed of four main components (Table 1): The supraspinous ligament (SSL), interspinous ligament (ISL), articular facet joint capsule (FJC), and ligamentum flavum (LF) [13].

Other spinal ligaments also exist, namely the anterior longitudinal, posterior longitudinal, intertransverse, and iliolumbar ligaments [13]. However, since none of these structures directly belong to the PLC, they will not be further discussed in this article.

Biomechanically, the PLC acts as a major stabilizer of the spine by forming a posterior tension band. Its main role is limiting flexion, axial rotation, and distraction of the spine, thereby increasing stability and reducing the axial load on the intervertebral discs [1,13,14]. As a result, injuries to the PLC are known to lead to spinal instability.

As defined by White and Punjabi, instability is “the loss of the ability of the spine under physiologic loads to maintain relationships between vertebrae in such a way that there is neither initial damage nor subsequent irritation to the spinal cord or nerve roots and, in addition, there is no development of incapacitating deformity or pain due to the structural changes” [2,15].

Classifications

The most frequently used classifications that directly concern PLC injuries in the setting of spinal trauma are the thoracolumbar injury classification system (TLICS) [5] and the AO Spine thoracolumbar classification [16]:

- The TLICS [5] is a point-based system for spinal trauma and includes three independent variables: 1) spinal fracture morphology, 2) PLC integrity, and 3) neurological status. In general, a score of 5 or more is an indication for surgery, 4 is indeterminate and depends on the surgeon’s preferences, and 3 or less is an indication for conservative treatment. Based on the TLICS, by solely documenting a PLC injury, three points would automatically be added to the score, whereas an indeterminate or an intact PLC would add two or no points, respectively.

- The AO Spine classification [4,6] depends on two variables: 1) fracture morphology where the status of the PLC is embedded within the fracture type and 2) neurological status. A clinical modifier has also been described pertaining to the cases where PLC status is inconclusive. However, this modifier remains useful mostly in patients with stable fractures where a PLC injury could drastically alter management.

As is evident in both the TLICS and AO Spine classifications, the status of the PLC plays a pivotal role in surgical decision-making, further highlighting the importance of properly diagnosing PLC injuries in subjects presenting with thoracolumbar trauma.

Injury patterns

Isolated spinal ligamentous injuries are rare owing to their superior tensile strength compared to bone. Therefore, most PLC injuries are associated with bony vertebral fractures [17].

Serious PLC injuries occur mainly with certain mechanisms including flexion-distraction or translation:

-Flexion/distraction injuries may be either purely osseous (osseous Chance or AO type B1) or an association of osseous and ligamentous injuries (osseoligamentous Chance or AO type B2), with purely ligamentous injuries being uncommon [6,17,18]. The most common mechanisms are seatbelt injuries, where prompt deceleration causes flexion of the trunk upon the seatbelt, with injuries generally located around the thoracolumbar junction. This leads to a horizontal split of the spine with the fracture line passing transversely through the vertebral body and pedicles, or solely through the intervertebral disk and facet joint, with any combination of the above being also possible. Associated intra-abdominal injuries are often found, prompting a careful examination of the abdomen with contrast CT scanning [19].

-Translation/displacement (AO type C) result from violent trauma and are characterized by displacement of the upper and lower parts of the spine around the fracture line in either the coronal, sagittal, or axial planes [6]. By definition, type C injuries are highly unstable with damage to the PLC being ubiquitously found [4,16]. In more severe cases, dislocation of the spine may occur with a high risk of associated life-threatening vascular compromise, severe neurological deficits [6], and other musculoskeletal injuries [20].

**Figure 2:**
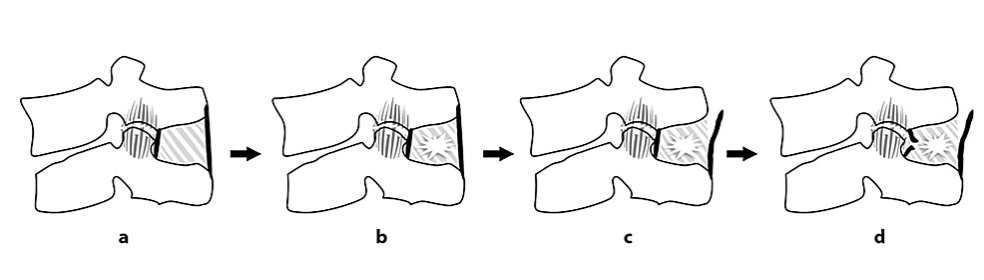
Sequential damage to the posterior ligamentous complex components: (a) A1/A2 fractures–FJC diastasis; (b) A3 fractures–FJC diastasis + ISL edema; (c) B2 fractures–FJC diastasis + ISL edema + SSL rupture; (d) B2/C fractures–Full PLC rupture ± facet fracture. FJC: Facet joint capsule, ISL: interspinous ligament, SSL: supraspinous ligament, LF: Ligamentum flavum

On the basis of the biomechanical properties of each component, sequential order of injuries (Figures 2a-2d) has been proposed by Pizones et al. [14].

- In compression fractures (AO type A1/A2), the PLC begins to fail at the level of the FJC and with a simple diastasis with fluid accumulation within the FJC. With more exaggerated flexion injuries (AO type A3), partial tears of the ISL, visualized as edema, are associated with FJC diastasis.

- In flexion/distraction injuries (AO type B2), SSL failure usually follows FJC diastasis and partial ISL tears. In more severe injuries where the tensile strain is applied even further, the ISL fails completely. The LF is generally the last structure to be damaged and succumbs when flexion/distraction forces are maximal.

- In translation injuries (AO type C), a complete ISL tear usually occurs, along with SSL and LF disruption. In these cases, the FJC may fail completely and even result in facet fractures or dislocations.

Of all the PLC components, the most important one to spinal stability has been shown to be the SSL. In fact, experimental studies have evaluated the effects of different components on spinal stability after stepwise resection of the ligaments, as proposed by Pizones (FJC, ISL, SSL, and finally LF). The results showed that FJC and ISL injuries did not significantly increase intervertebral range of motion compared to SSL injuries. As a result, the SSL is thought to be the main stabilizing component of the PLC, and rupture of this structure is thought to lead to PLC incompetence and spinal instability [8,21,22]. 

Diagnosis

Physical Exam

Following hemodynamic stabilization, the diagnostic process starts with a thorough physical exam. Clothing should be removed while considering the risk of hypothermia. Any bruising on the chest or abdomen in areas of the seatbelt, known as the seatbelt sign, is highly suggestive of flexion/distraction injuries. The log roll maneuver is then used to safely turn the patient sideways, allowing for the examination of the spine. Any signs of bruising, edema, or visible swelling coupled with tenderness on palpation of the spinous processes should alert the examiner to a probable underlying spinal fracture. If a step-off is palpated with increased distance between subsequent spinous processes, a PLC injury is likely [23]. The physical exam should then be completed with a thorough neurological evaluation according to the American Spine Injury Association (ASIA) classification, as neurological deficits increase the likelihood of PLC injury and spinal instability [24]. Even with a meticulous physical examination, up to 20% of thoracolumbar injuries may be missed, especially in subjects presenting with multiple injuries or altered levels of consciousness [20]. This is particularly true due to the high-velocity nature of these traumas. 

Medical Imaging

**Figure 3:**
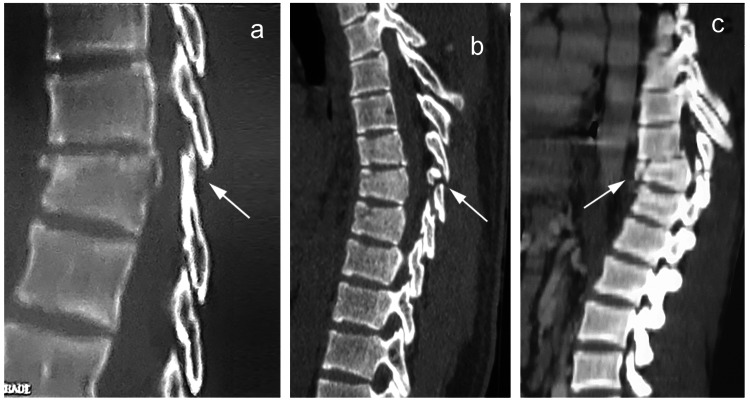
Indirect signs of posterior ligamentous complex injury on sagittal CT scans: (a) splaying of the spinous processes, (b) avulsion of the spinous process, and (c) translation of the vertebrae with associated fracture.

Various imaging modalities may be used for the diagnosis of PLC injuries, including conventional radiographs, CT scans, and MRI. With both conventional radiographs and CT-scans, the soft tissues cannot be directly visualized, and PLC injuries may be inferred by the presence of certain telltale findings (Figures 3a-3c) such as splaying of spinous processes, avulsion of superior or inferior margins of spinous processes, widened facet joints, empty (“naked”) facet joints, perched or dislocated facet joints, and vertebral translation or rotation [25].

**Figure 4:**
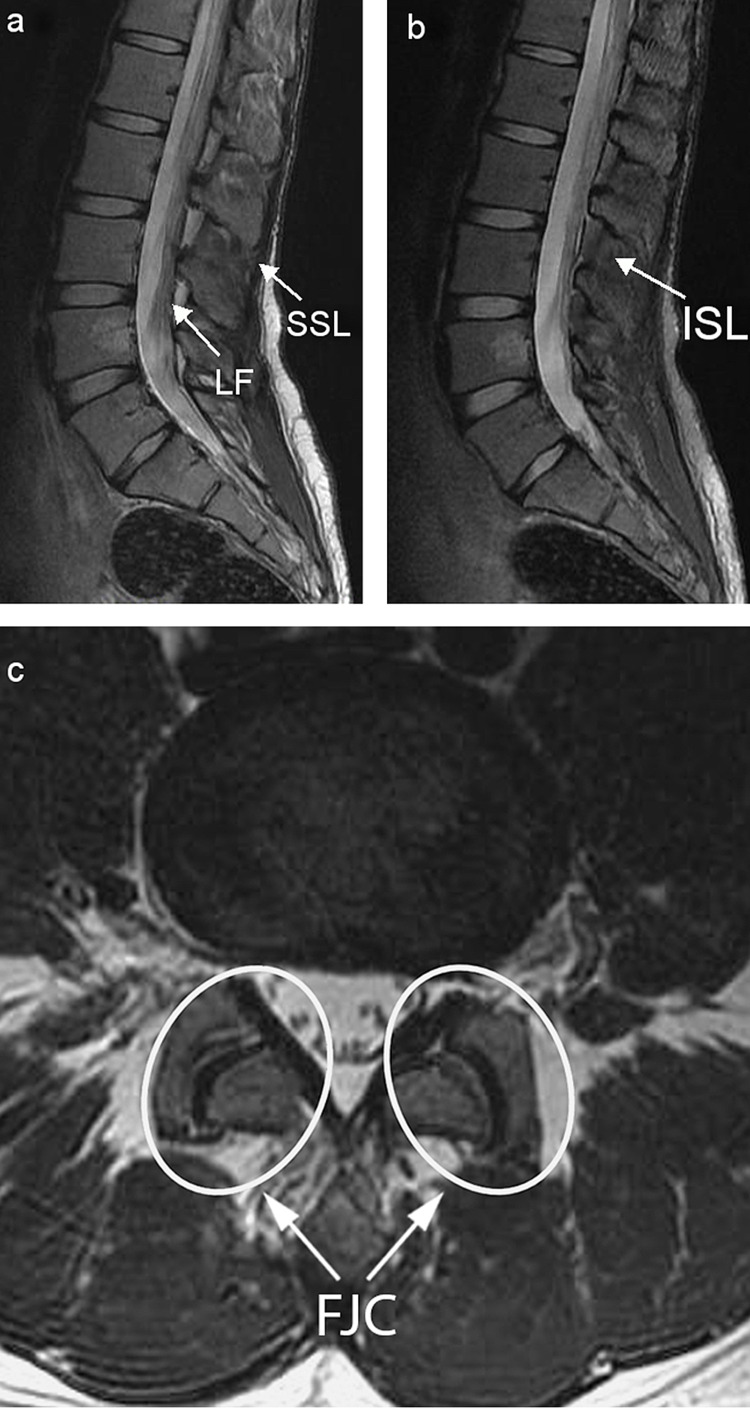
(a) Sagittal T2-weighted lumbar MRI: SSL, LF. (b) Sagittal fat-saturation T2-weighted lumbar MRI: ISL. (c) Axial fat-saturation T2-weighted lumbar MRI: FJC. SSL: supraspinous ligament, LF: ligamentum flavum, ISL: interspinous ligament, FJC: facet joint capsule

Purely osseous findings, such as loss of vertebral body height or local kyphosis, are unreliable and should not be used as signs of PLC injury [26]. In this regard, radiographs and CT scans lack precision, and MRI remains the gold standard modality for the assessment of PLC integrity with high global sensitivity for PLC injury detection of around 90% [27,28]. This imaging modality allows the direct visualization of soft tissues around the vertebrae. Each element of the PLC is best analyzed on different sequences (Figures 4a-4c) [5,8,27,29-31].

**Table 1:** Anatomical description of the four components of the posterior ligamentous complex. PLC: Posterior ligamentous complex

Ligament	Description
Supraspinous ligament (SSL)	Runs along medial line, attaches to tips of spinous processes; Most posterior structure of the PLC and is the first ligament encountered in posterior surgical approaches to the spine
Interspinous ligament (ISL)	Runs parallel to spinous processes and connects upper and lower processes across their entire lengths; Extends from LF anteriorly to SSL posteriorly
Facet joint capsules (FJC)	Ligament around superior and inferior facets of adjacent vertebrae; Four FJC at each vertebral level: two cranially and two caudally
Ligamentum flavum (LF)	Connects laminae of adjacent arches, is the most anterior structure of the PLC; Originates from anterior surface of upper lamina and ends at superior portion of lower lamina

- Sagittal T1- and T2-weighted images are best to evaluate the LF and SSL.

- Sagittal STIR and fat-saturation T2-weighted sequences are best to evaluate the ISL.

- Axial fat-saturation T2-weighted sequences are best to evaluate the FJC.

Even though the LF and SSL on MRI are categorized binarily into either “intact’ or” “ruptured,” a gray area of “indeterminate” findings is encountered in the case of the FJC and ISL, owing to findings of edema on MRI. Certain signs allow for the proper evaluation of the PLC [30]:

- Discontinuity of the black stripe visualized on sagittal T1- and T2-weighted images signifying LF or SSL tears.

- Hyper-intense fluid signals on sagittal STIR or fat-saturation images signifying an ISL tear.

- Hyper-intense fluid signals in facet capsules on axial fat-saturation T2-weighted sequences signifying capsular injury.

While MRI is mandatory for patients with neurological deficits [20], the superiority of this imaging modality for the detection of PLC injuries does not justify its widespread use in all patients presenting with thoracolumbar fractures. In fact, MRI has shown a tendency to overestimate ligamentous injury [32,33], especially in subjects with no or mild neurological compromise (ASIA D and E), where the accuracy of ligamentous injury on MRI compared to subjects with a more severe state (ASIA A to C) is significantly decreased [28]. Applying more stringent criteria for the evaluation of PLC injuries on MRI should decrease the risk of overdiagnosis [34]. Furthermore, a recent study suggested that patients without neurological deficits who were assessed using CT-scans, and where no indirect signs of PLC injury were found did not require routine MRI evaluation if non-operative treatment was planned, since doing so rarely changed clinical management. In this study, the authors also suggested that, for neurologically intact patients where surgical management is considered, MRI is recommended as it may change the treatment strategy [35]. However, where a ligamentous injury is clinically suspected with negative x-rays and/or CT scans, an MRI must be obtained.

Treatment

Different treatment options exist for thoracolumbar injuries, ranging from non-surgical treatments to instrumentation and spinal fusion. The management usually depends on a wide range of variables, of which spinal stability and neurological status reign supreme [7,10,16,36,37].

Non-operative Treatment

Stable fractures may be treated non-operatively. This includes purely osseous spinal injuries where healing and recovery are generally straightforward. Nevertheless, strict clinical and radiographic follow-up is necessary. Whenever PLC injuries are found, the clinical picture becomes more complicated due to the inherent instability conferred by the PLC injury, and no clear consensus is found on the optimal treatment method [20]. Nonetheless, what is certain is that there is no place for conservative management in PLC injuries [38,39].

Recently, algorithms have been proposed for the operative and non-operative treatment decision-making in spinal trauma [10,11].

Operative Treatment

Injuries of the PLC are thought to present difficulties during the healing process. Without surgery, the natural history of an injured PLC leads to spinal instability, progressive kyphotic deformity, and subsequent vertebral fracture collapse. In order to counter these risks, spinal fractures are commonly treated by synthesis, PLC injuries by spinal fusion [17,40-43], and neurological compromise by decompression [20]. While ligamentous injuries in other parts of the body (e.g. Achilles tendon) are known to heal after continuity has been restored [8,44], healing of the PLC has not been established. A recent study showed that, after reducing the gap in PLC injuries of the cervical spine, ligamentous healing may be achieved in most patients without the need for spinal fusion [45]. Nonetheless, further studies must assess this claim in thoracolumbar PLC injuries and of varying types and severity. 

As for spinal fixation, many options and variations exist, including posterior, anterior, or combined approaches with long or short segment fixations, with or without instrumentation of the fractured vertebra [9,39]. No matter the construct, the primary goal of fixation remains stabilization of the spine to allow for proper fracture healing. Detailed discussions about decision-making for the type of fixation are beyond the scope of this article.

Spinal Instrumentation and Fusion 

**Figure 5:**
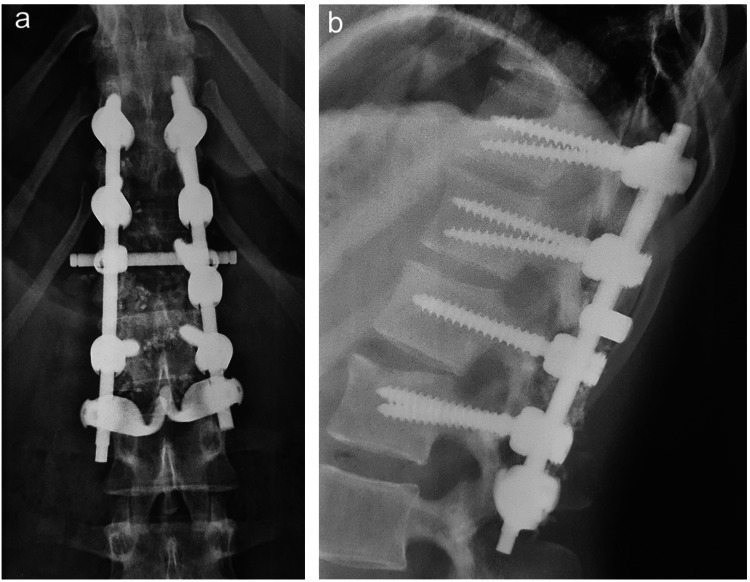
Postoperative (a) anteroposterior and (b) lateral radiographs of the lumbar spine with instrumented fusion from T11 to L2.

Fusion entails the placement of bone or other biologically active substances around the spine in order to fuse segments, provide stability, and promote healing [46]. Since PLC injuries are considered highly unstable, fusion aims to increase stability (Figures 5a, 5b). As a result, spinal fusion remains the gold standard for PLC injuries, as the lack of PLC healing constitutes a major risk for nonunion and secondary sagittal deformity [21].

Historically, all spinal injuries requiring surgery were fused, due to reports of instrument failure without the added support of fusion [47]. However, fusion has shown increased surgical times, blood loss, and need for transfusion. This process may also require bone graft harvesting from the posterior iliac crest, an act associated with lingering pain even years postoperatively [46,48-50]. Moreover, spinal fusion is associated with decreased spinal flexibility and segmental motion, leading to higher patient dissatisfaction scores [12,51]. This increased stiffness renders the region above the fusion site the last mobile segment, thereby leading to degenerative changes and adjacent segment disease [52]. Fusion is therefore avoided when possible, especially in younger populations, due to the increased lifelong risk of adjacent segment disease, or in the pediatric population where the spine must be allowed to grow [53]. In addition, many of the studies showing the inadequacy of spinal instrumentation alone were conducted decades ago, with older, less stable constructs than what is available today [47]. 

Spinal Instrumentation Without Fusion (Internal Bracing)

**Figure 6:**
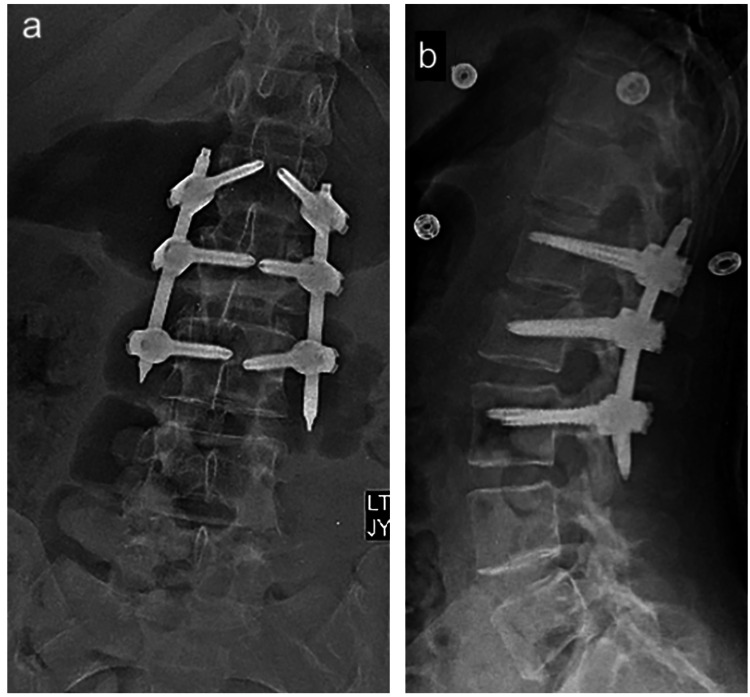
Postoperative (a) anteroposterior and (b) lateral radiographs of the lumbar spine with short-segment fixation without fusion from L1 to L3.

The thoroughly studied burst fractures demonstrated identical results between fusion and non-fusion groups [34,48,49,54-57]. As a result, surgeons have been increasingly evaluating the efficacy of spinal instrumentation without fusion in other unstable fracture types (Figures 6a, 6b). The rationale behind this approach in PLC injuries is similar to that of laminectomy without instrumentation. Reducing the fractured vertebral kyphosis and stabilizing it with temporary fixation - a sort of internal bracing of the spine - could allow healing of the fracture. These types of constructs are generally temporary, and instrumentation is usually removed after achieving bony healing. This allows the restoration of spinal motion and flexibility [58]. As a result, even if no direct PLC healing has been achieved, the previously unstable fracture would transform into a laminectomy model. In fact, one might further argue that a usual laminectomy might be even more detrimental to spinal stability than a traumatic PLC injury, as not only is the PLC resected in its entirety, but so are the laminae and part of the facet joints. Moreover, a recent study showed that a single-level laminectomy might not cause instability of the spine [59]. As such, it appears that, even in spinal fractures with PLC injuries, temporary instrumentation of the spine until complete healing of the fracture may be a valid treatment option.

With the advent of minimally invasive spine surgery (MISS), the length of surgery, paraspinal soft tissue dissection, and bleeding have been even more reduced [12,40,60-63]. Posterior percutaneous approaches allow for instrumentation and lateral approaches may be used for corpectomy in cases where anterior column support is needed [63-65].

While burst fractures have been thoroughly studied, limited data exist on the fusion less treatment of more unstable fractures, including those presenting with PLC injuries (AO types B and C). Most studies on patients with osseoligamentous flexion/distraction injuries treated by temporary fixation without fusion, including MISS, have yielded positive results [46,65]. One study by Kim et al. [66] on subjects with flexion/distraction fractures with PLC injury showed favorable results for instrumentation without fusion, with little loss in vertebral height or progressive kyphosis. However, this study consisted of a select group of young patients and lacked a control group. Another study by Grossbach et al. [38] found similar results in an open instrumented fusion group compared to MISS in subjects with flexion/distraction injuries. Kocanli et al. [67] treated all spinal fracture types (AO types A, B, and C), regardless of stability, with instrumentation without fusion and found generally favorable results even 10 years post-surgery, leading the authors to suggest treating most spinal fractures without fusion. This study also did not include a control group. While these results are very encouraging, more studies must be conducted in order to make a stark conclusion on this matter.

**Figure 7:**
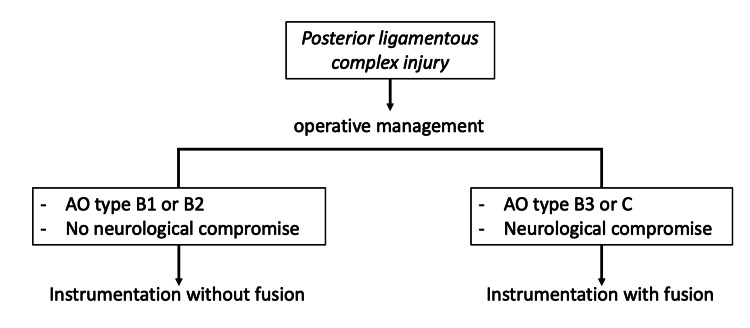
Algorithmic approach to the management of posterior ligamentous complex injuries.

An algorithm is presented in Figure 7 based on the authors' approach to the management of PLC injuries.

Spinal Instrumentation Removal

Hardware removal is controversial since many variables must be considered, of which the most paramount is whether fusion was undertaken or not, and whether the primary injury was purely osseous or associated with PLC injuries. A review of the literature only muddles the picture, as most articles have either studied only burst-type fractures or have come to broad conclusions without reporting on PLC status.

Some authors have reported feelings of foreign body or back pain attributed to the hardware, though these cases were rare [58,68-70]. Conversely, it appears that the main advantage of hardware removal after fracture healing would be the restoration of spinal mobility.

While some formal indications exist for the removal of spinal instrumentation, elective removal remains controversial, with some of the more widely accepted indications being instrument failure or loosening, infection, protruding hardware, and screw misplacement [58]. In fact, in patients treated with spinal fusion, hardware-related back pain does not seem to subside [71] and no gain in mobility has been recorded after hardware removal [46,72]. Moreover, the extensive exposure and tissue destruction required during the act of fusion, including any remaining posterior ligaments and other stabilizing tissues, may lead to progressive kyphotic deformity if the hardware is later removed [51,73]. Therefore, in these subjects, hardware removal should not be routinely indicated.

**Figure 8:**
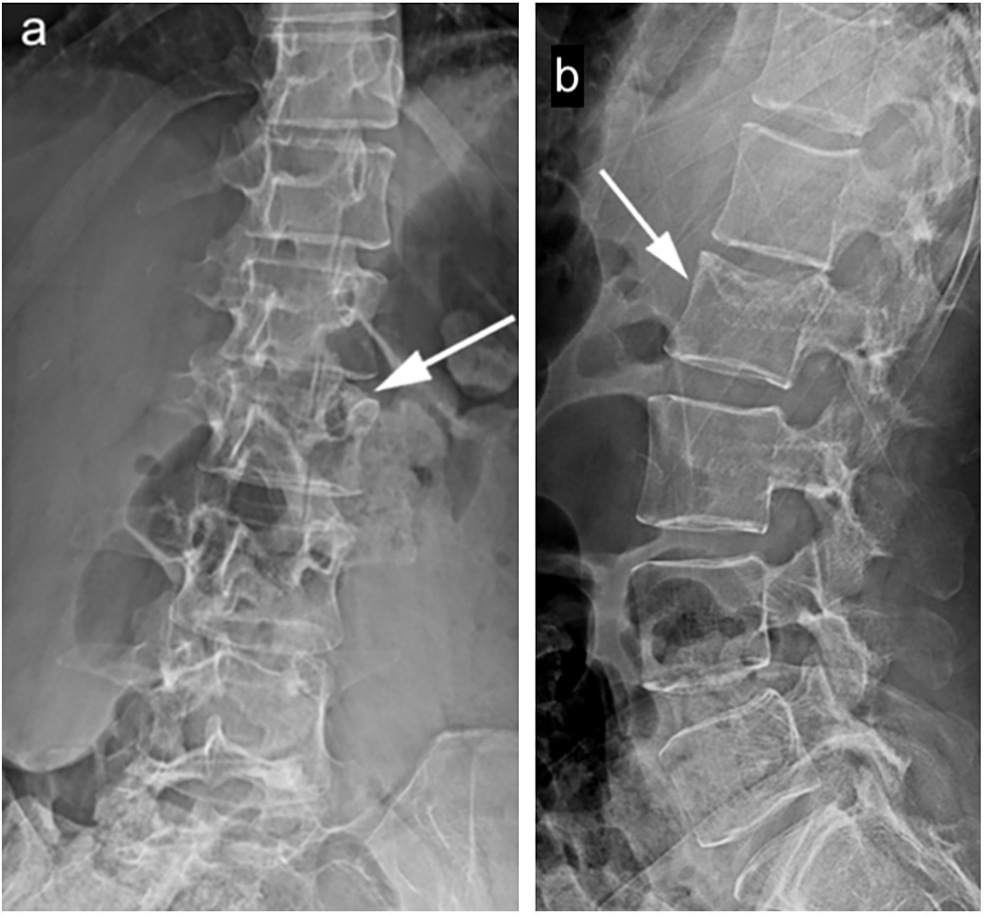
(a) Anteroposterior and (b) lateral radiographic follow-up 11 months after hardware removal in the same patient as Figures 6a, 6b showing no increased kyphosis.

The real debate remains in patients operated without fusion, where the choice of hardware removal is less well defined. For some surgeons, fear of instrument failure due to hardware fatigue is considered enough reason to routinely remove the hardware after the fracture has healed [51]. Furthermore, removal of hardware in these patients restores spinal mobility, though primarily at the level of the lumbar spine, since a thoracic range of motion is already limited [46,72]. The picture is further distorted in the presence of PLC injuries. As these ligamentous injuries may not heal effectively, the safety of hardware removal after bony healing may be questioned. However, there is increasing evidence that thoracolumbar injuries with a ruptured PLC may be effectively treated by temporary spinal fixation without fusion, with little to no progressive kyphosis or sagittal imbalance being reported up to 10 years after hardware removal (Figures 8a, 8b) [38,46,53,66,74].

As for the timing of instrument removal, this topic remains controversial and little data is available in subjects with PLC injuries, as most studies either do not indicate a time frame or do not differentiate between subjects with and without PLC injuries. Although Grossbach et al. [38] do not recommend the routine removal of hardware, the authors proposed a 12-month mark without any supporting evidence. Maintaining hardware in place may thus present a viable option in patients treated with fixation without fusion, especially those whose fixation was primarily at the level of the thoracic spine, as this group of patients will not significantly benefit from hardware removal [46,72]. One study by Kim et al., in which all patients who presented with PLC injuries and underwent instrumentation without fusion and were later operated on for removal of hardware in a time frame ranging between 6 and 16 months with an average of 9.7 months, found satisfactory results in most patients [67]. In all cases, a CT scan of the fractured segment should be obtained prior to removal in order to assess bony healing [46].

## Conclusions

Injuries of the PLC are often missed in the setting of thoracolumbar trauma and may lead to serious complications if not discovered in due time. These injuries may be suspected on clinical exam, and the diagnosis is often made either indirectly by x-rays and CT scanning, or directly via MRI. The imaging modality of choice remains the MRI and is mandatory in patients with high suspicion of PLC injuries. Surgical management is the norm in these injuries, as PLC damage, especially coupled with fractured vertebrae, renders the spine unstable. Although fusion was once the standard of care in these patients, recent endeavors seek to avoid this, except in certain patients, and temporary spinal fixation without fusion is increasingly gaining traction in patients with PLC injuries.
